# Study on Thermal Conductivity of P-Phenylenediamine Modified Graphene/Epoxy Composites

**DOI:** 10.3390/polym14173660

**Published:** 2022-09-03

**Authors:** Jun Lin, Jiancheng Zhou, Mengyao Guo, Danqing Chen, Guohua Chen

**Affiliations:** Xiamen Key Laboratory of Polymers & Electronic Materials, College of Materials Science and Engineering, Huaqiao University, 668 Jimei Blvd, Xiamen 361000, China

**Keywords:** p-phenylenediamine, graphene, epoxy

## Abstract

Thermal management has become an important requirement for many types of electrical equipment due to the development of integrated circuits. In this study, modified and reduced graphene fillers were synthesized in two steps, and then epoxy resin was filled through the evaporation of the solvent. The interfacial thermal resistance between the filler and matrix material was lowered by including amino groups to improve graphene compatibility in the epoxy resin. Furthermore, the reduction procedure was shown to have the potential to fix graphene oxide flaws, thereby improving thermal stability, electrical conductivity, and thermal conductivity of the composites. As a result, the thermal conductivity of the composite reached 1.7 W/mK, which is 750% higher than that of pure epoxy resin, and it was still insulated.

## 1. Introduction

With the rapid development of electronic equipment in modern society, the chip integration of various types of electronic equipment is improving rapidly [1]. According to Moore’s Law, the packaging density of electronic devices doubles every one and a half years. Therefore, solving the thermal management problem of electronic devices has become a top priority to ensure the service life of electronic devices [2]. Epoxy resin (EP) is widely used in printed circuit boards, electronic packaging materials, chemical heat exchangers, and other fields because of its advantages of good mechanical properties, low shrinkage, good solvent resistance, low cost, and easy processing. However, it has poor thermal conductivity, and it cannot dissipate heat efficiently [3]. To improve the thermal conductivity of EP, researchers have tried to add various particles with high thermal conductivity into the EP matrix such as metal fillers, including Ag nanoparticles [4], Cu nanowires [5], ceramic fillers (such as SiO_2_ [6,7], AlN [8,9], and BN [10,11]), and carbon-based materials (such as diamond [3], carbon nanotubes [12], and graphene [13]). Graphene, particularly, is considered to be an ideal material for thermal conductivity particles due to its extremely high thermal conductivity [14].

However, the poor dispersion of graphene and its incompatibility with epoxy resin have been the main problems restricting the development of a composite. Hence, in order to improve the dispersion of graphene and the matrix material, researchers have used various methods to prepare a three-dimensional graphene thermal conductivity network. For example, Gong et al. [15] used chemical vapor deposition (CVD) technology in graphene nickel net growth, with a strong acid-etching metal nickel, leaving the graphene 3D mesh structure and polyimide composite film. Although CVD technology can guarantee the quality of graphene, its low yield and complex technology are not suitable for industrial large-scale production. Lian and others [16] experimented with the use of a special cooling liquid oxidation directional solidification graphene in a three-dimensional network structure, the final epoxy resin composite. The three-dimensional structure of graphene produced in this way is unstable and cannot be guaranteed to remain three-dimensional in the composite material. Alam et al. [17] coated graphene onto the surface of polypropylene and extruded it into a three-dimensional connected structure by hot pressing. However, the graphene load of this method cannot be significantly increased, which restricts the further improvement of its performance. On the other hand, in order to improve the interface compatibility between graphene and matrix materials, researchers synthesized functionalized rGO by covalent or non-covalent modification and reduction methods, taking advantage of the fact that GO is rich in hydroxyl, carboxyl, and epoxy groups, as well as other groups, which can be easily modified. For example, Li et al. [18] grafted polyaniline (PANI) onto the surface of rGO aerogel. Liu et al. [19] modified reduced graphene oxide (rGO) with polytriazine to improve the dispersion and interfacial interaction of graphene in bismaleimide (BMI) resin. Wang et al. [20] modified graphenes with the surface modifier poly4-styrene sodium sulfonate (PSS) to prevent the aggregation of graphene in polyvinyl alcohol (PVA). Liu et al. [21] grafted hydroxyl graphene to 3-methacrylate oxypropyl trimethoxysilane (KH-570) to ensure its good dispersion and interfacial compatibility in the polydimethylsiloxane (PDMS) matrix. When the amount of covalently modified filler is 2 wt%, the thermal conductivity of the composite reaches 0.761 W/mk. These modification methods through various modifiers can not only improve the properties of graphene but also easily control the amount of filler used in the composite. However, other defects are inevitably introduced in the modification process, which restricts the improvement of the composite’s properties.

Due to the presence of amino groups in p-phenylenediamine, there is a certain amount of reduction of GO, and the interface compatibility between graphene and epoxy resin can be improved by grafting amino groups. Therefore, we used p-phenylenediamine as a modification material to covalently modify GO and then reduced the modified PPD-GO to repair the defects. It was eventually filled into the epoxy resin by evaporation of the solvent. Infrared and XPS tests were used to explore the reduction effect of filler modification, Raman tests were used to explore the defects of filler, and SEM and thermal conductivity tests were used to explore the relationship between the interface of composite materials and thermal conductivity.

## 2. Materials and Methods

### 2.1. Materials

Natural flake graphite (150 mesh; Purity > 99%) was provided by Qingdao Tianhe Graphite Co., Ltd. (Qingdao, China). P-phenylenediamine (PPD, AP), potassium permanganate (KMnO_4_, AP), ascorbic acid (VC), sulfuric acid (H_2_SO_4_, 98%) and hydrogen peroxide (H_2_O_2_, 30% AQ.) were purchased from Sinopharm Chemical Reagents Co., Ltd. (Shanghai, China). Diethyl toluene diamine (DETDA) was purchased from Jinan Noshi New Materials Co., Ltd. (Jinan, China). Table 1 shows the physicochemical characteristics of the matrix material at room temperature.

### 2.2. Preparation of GO Modified by P-Phenylenediamine

**Figure 1:**
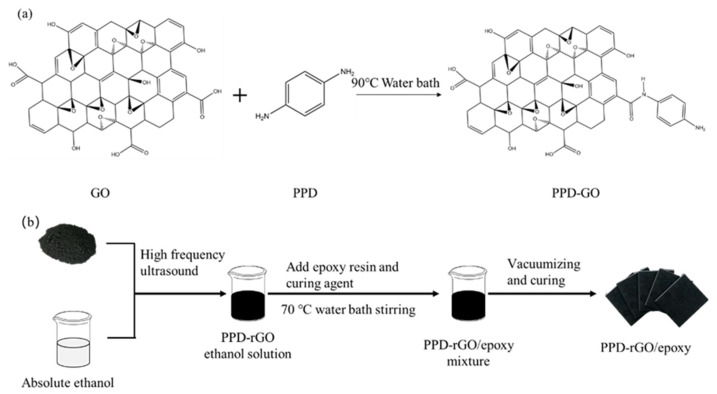
(**a**) Structure diagram of PPD-modified GO. (**b**) PPD-rGO epoxy composite preparation flow chart.

The modified Hummers method was used to oxidize natural flake graphite into graphite oxide (GO) [22] at room temperature. GO was dissolved in 250 mL ultra-pure water and dispersed evenly by high-frequency ultrasound. Then, appropriate PPD (GO:PPD = 1:10) was added. Then, ammonia was added until PH reached 11. The modification mechanism was completed by stirring for 3 h in a water bath, as shown in Figure 1a. The carboxyl group on GO and the amino group on p-phenylenediamine were aminated so that PPD was covalently grafted to GO, and the amino-functionalized modified GO was obtained, named PPD-GO.

### 2.3. Reduction of PPD-GO by Ascorbic Acid

An appropriate amount of ascorbic acid (GO:VC = 1:10) was added to the PPD-GO solution, stirred in 80 °C water baths, condensed and refluxed, and named PPD-rGO after 8 h of reaction. The PPD-rGO solution after the reaction was extracted and filtered while hot and washed repeatedly with hot water and ethanol until the filtrate was nearly colorless, and then PPD-rGO was freeze-dried for later use.

### 2.4. Preparation of PPD-RGO Epoxy Composites

The preparation process is demonstrated in Figure 1b. An appropriate amount of anhydrous ethanol was added to PPD-rGO, and then it was evenly mixed by high-frequency ultrasound. Epoxy resin was added and stirred in 70 °C water baths for 30 min.

### 2.5. Characterization

The samples were characterized using NICOLET iS 50 INFRARED spectrometer, in Via confocal Raman spectrometer, Thermo Fisher Scientific K-Alpha + X-ray photoelectron spectrometer, thermogravimetric analyzer (TGA-50H), and scanning electron microscope. The powder samples were prepared by compression method and their spectra of 500–5000 cm^−1^ were measured on an IR spectrometer. Raman spectrograms were recorded at wavenumber of 1000–20,000 cm^−1^. The X-ray photoelectron spectrometer uses a monochrome Al K-α with energy of 1486.68 eV. A thermogravimetric analyzer was used to measure from 30 °C to 800 °C in an air atmosphere. The thermal conductivity of the composite was measured by LFA 447. The volume resistivity of the composite material was measured by an insulation resistance meter (ZC36) and a Keesley 2400 digital source meter.

## 3. Results and Discussion

**Figure 2:**
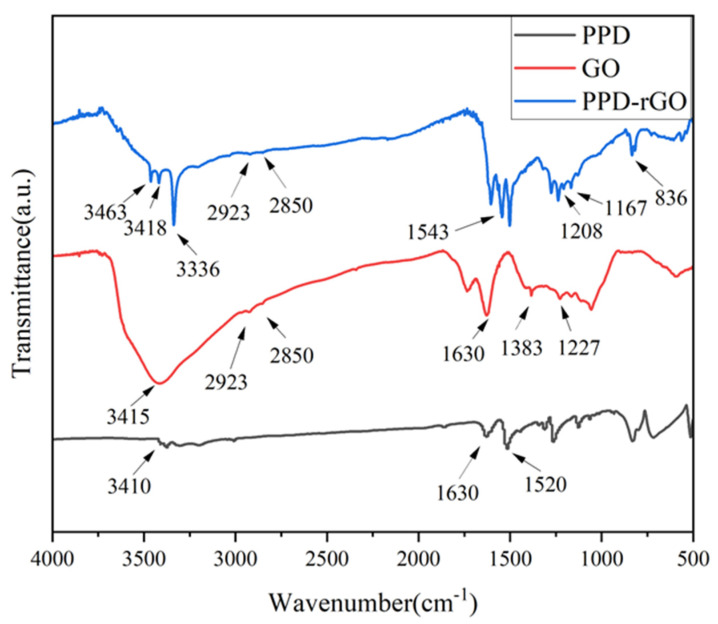
FT-IR spectra of PPD, GO and PPD-rGO.

Infrared characterization was carried out to investigate the group grafting of P-phenylenediamine-modified GO. Figure 2 shows the infrared spectra of GO and PPD-rGO. By comparison, we found that the infrared spectra of PPD, GO and modified and reduced PPD-rGO were significantly different. In the infrared spectrum of PPD, the two peaks around 3410 cm^−1^ were the -N-H stretching vibration of NH_2_. The peaks at 1620 cm^−1^ and 1520 cm^−1^ were attributed to the N-H bending vibration of NH_2_. In the infrared spectra of GO, it was observed that there was a wide-stretching vibration peak of hydroxyl at 3415 cm^−1^. The 2923 cm^−1^ and 2850 cm^−1^ peaks correspond to the antisymmetric and symmetric stretching vibration peaks of saturated CH_2_ in GO, respectively. At 1630 cm^−1^ was the stretching vibration peak of the C=C skeleton of a carbon atom bonded by a benzene ring on GO in *sp*^2^ hybridization mode. The absorption peak near 1383 cm^−1^ was the stretching vibration of C-O in the carboxyl group. Compared with PPD-rGO, the absorption spectra at 3463 cm^−1^ and 3418 cm^−1^ were attributed to the stretching vibration of the N-H of primary amine. The stretching vibration peak of the hydroxyl group at 3336 cm^−1^ became narrower and smaller due to modification and reduction. The antisymmetric and symmetric stretching vibration peaks of saturated CH_2_ at 2923 cm^−1^ and 2850 cm^−1^ remained unchanged. The stretching vibration peaks of C-N and N-H in the vicinity of 1167 cm^−1^ and 836 cm^−1^ corresponded to stretching vibration peaks of C-N and N-H. It is worth noting that, due to the amidation reaction between the carboxyl group on GO and the amino group on PPD, the absorption peaks near 1543 cm^−1^ and 1208 cm^−1^ corresponded to the amide II band and amide III band, respectively [23]. Therefore, it can be proved that PPD is covalently grafted to GO, and then PPD-rGO is obtained through reduction.

**Figure 3:**
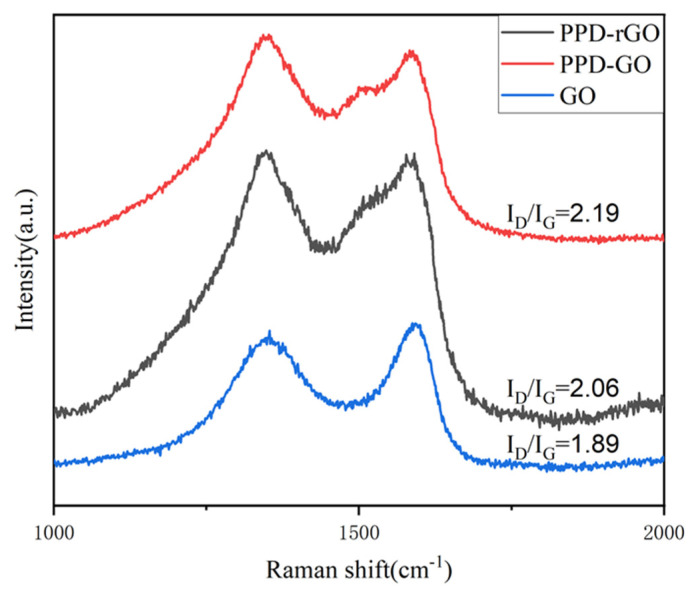
Raman shift of GO, PPD-Go, and PPD-rGO.

The Raman test was used to explore the defects in modified and reduced GO. Figure 3 is the Raman shift of GO PPD-GO and PPD-rGO. The ratio of I_D_/I_G_ is calculated by the fitting peak area. The D peak is the defect peak, and the G peak reflects the symmetry and crystallization degree of graphene, as can be seen from the figure; after the covalent modification of GO by PPD, its I_D_/I_G_ increased from 1.89 to 2.19, indicating that the covalent modification led GO to introduce other groups to increase its defect degree, while the I_D_/I_G_ of PPD-rGO reduced by VC decreased to 2.06, which repaired some defects to a certain extent. The figure shows that g-peak splitting occurs in both PPD-GO and PPD-rGO. The reason for this is that the presence of randomly distributed amines affects the charge distribution on the surface of GO, and the local vibration mode of amines interacts with the phonon mode of GO, resulting in splitting, which means chemical grafting of PPD occurs on the surface of GO [24].

**Figure 4:**
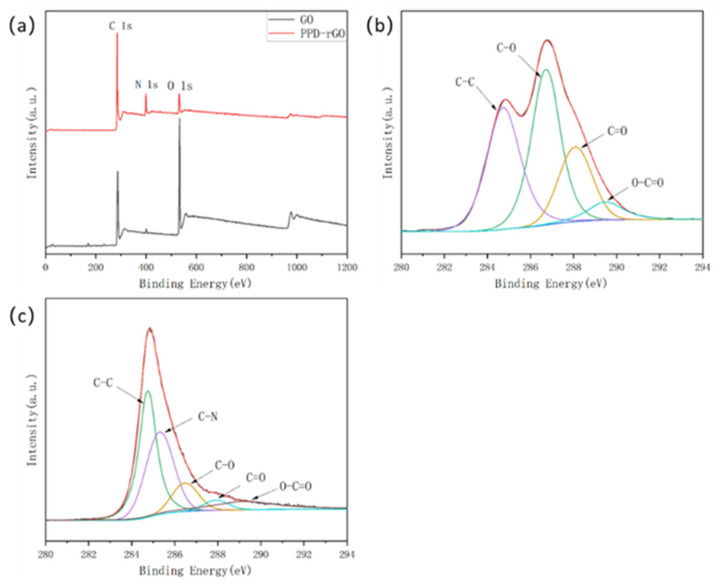
XPS spectra of GO and PPD-rGO. (**a**) Full spectrum; (**b**) C 1s spectrum of GO; (**c**) C 1s spectrum of PPD-rGO.

To further explore the changes in functional groups in the samples, an XPS characterization of GO and PPD-rGO was carried out. It can be seen from the full spectrum that there are mainly C and O elements in GO. In addition to C and O elements, there was an N element from p-phenylenediamine modified in PPD-rGO after modification and reduction, and O element also decreased sharply in the process of modification and reduction. Figure 4b,c are the c 1s sub-peak diagrams of GO and PPD-rGO, respectively. It can be seen from the figure that GO and PPD-rGO had the same peak, and the binding energy values were 284.8 eV, 286.8 eV, and 288.1 eV, respectively. The characteristic peaks of C-C, C-O, C=O, and O-C=O correspond to 289.4 eV, while the C-N peak of 285.6 eV exists in PPD-rGO, which belongs to the c-n bond generated after the amidation of p-phenylenediamine modified GO, indicating that the modification and reduction were successful [25,26].

**Figure 5:**
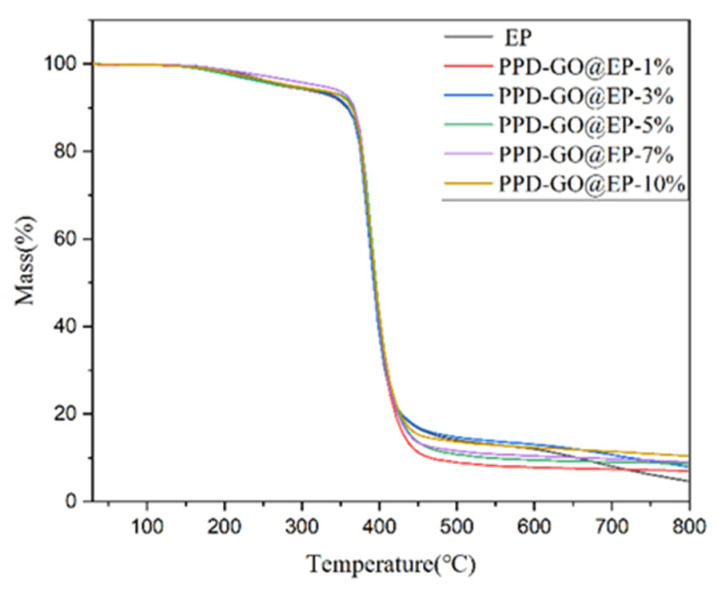
Thermogravimetric curves of composite materials.

Figure 5 shows the thermogravity diagram of the composite material, and Table 2 shows the characteristic thermal parameter table of the composite material. Figure 5 highlights the fact that when the temperature rises to about 200 °C, hydrophilic groups such as the hydroxyl carbonyl group in the epoxy material begin to decompose, and when the temperature rises to 350 °C, the cross-linking network of the epoxy resin begins to decompose. Due to the addition of thermally conductive filler with an amino group, the interface compatibility of epoxy resin and filler is improved, and the physical and chemical crosslinking points between the filler and matrix are increased. The corresponding thermal decomposition temperature T10% of mass loss is higher than that of pure epoxy resin T10%, and the final residual amount also increases with the increase in the mass fraction of filler, so the thermal stability of the composite is improved.

**Figure 6:**
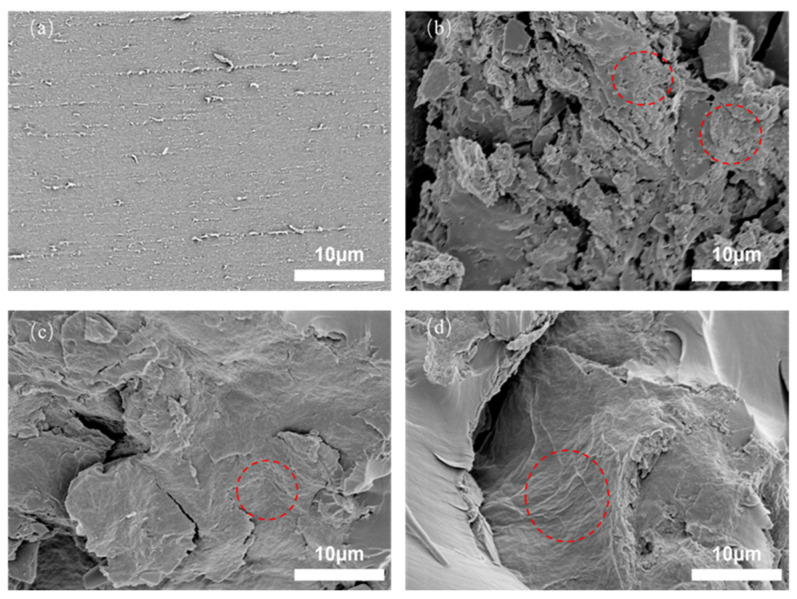
SEM images of the fracture surface for (**a**) pure EP, (**b**) rGO/EP, (**c**) PPD-Go /EP, (**d**) PPD-rGO/EP composites.

The interface compatibility between epoxy resin and filler was investigated using SEM. Figure 6a is the SEM image of pure epoxy resin. It can be seen that the surface was relatively smooth except for partial scratches; however, the cross-section of the composite was different in terms of roughness after filling. Figure 6b is the cross-section of unmodified rGO-filled epoxy resin. It can be seen that there was a high level of roughness at the cross-section due to the poor compatibility between rGO and epoxy resin. Figure 6c is the cross-section of the modified PPD-GO-filled epoxy resin. It can be seen that due to the lack of good compatibility between rGO and epoxy resin, there was a high level of roughness in this section. Figure 6c is the cross-section of the modified PPD-GO-filled epoxy resin. It can be seen that the compatibility of the cross-section was improved to some extent, indicating that the modification made the amino group of the GO band react with the epoxy group in the epoxy resin, which improved the compatibility between the filler and the matrix and prevented agglomeration. Figure 6d is the composite material of modified and reduced PPD-rGO and epoxy resin. Its cross-section is similar to Figure 6c. It can be concluded that reduction does not change the functional results of amino groups, but only plays a role in eliminating part of the oxygen-containing groups and repairing defects.

**Figure 7:**
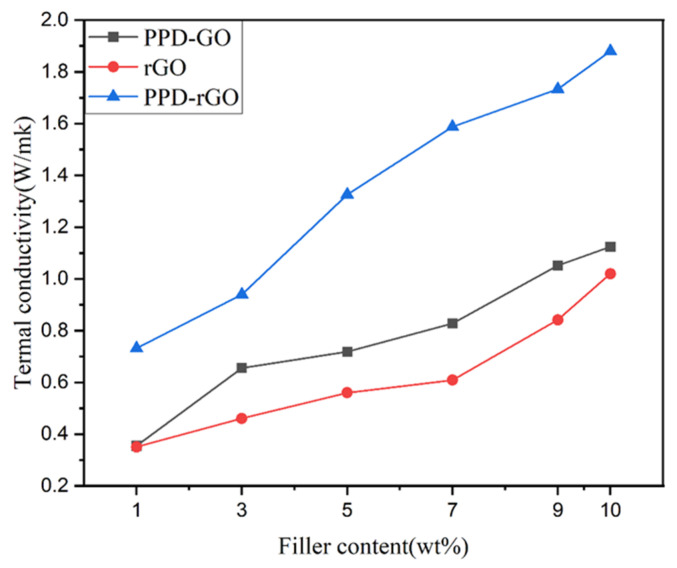
Thermal conductivity diagram of composite materials.

Figure 7 shows the thermal conductivity of composite materials. In this figure, the thermal conductivity of rGO/EP and PPD-GO/EP increases with the increase in mass fraction. By comparing the thermal conductivity of the two, it was found that the GO modified by p-phenylenediamine alone has a greater impact on the thermal conductivity of the composite than the GO reduced by VC alone. However, rGO may exist in a small range of agglomerations in the matrix material, which may be caused by its isolated existence in a small range and the insufficient construction of the thermal conduction path. Therefore, the modified and reduced PPD-rGO not only has an amino group that is compatible with the epoxy resin matrix, but also repairs its defects to a certain extent. Therefore, when the amount of the filler reaches 9 wt% and 10 wt%, the thermal conductivity of the composite reaches 1.7 W/mK and 1.88 W/mK, respectively.

**Figure 8:**
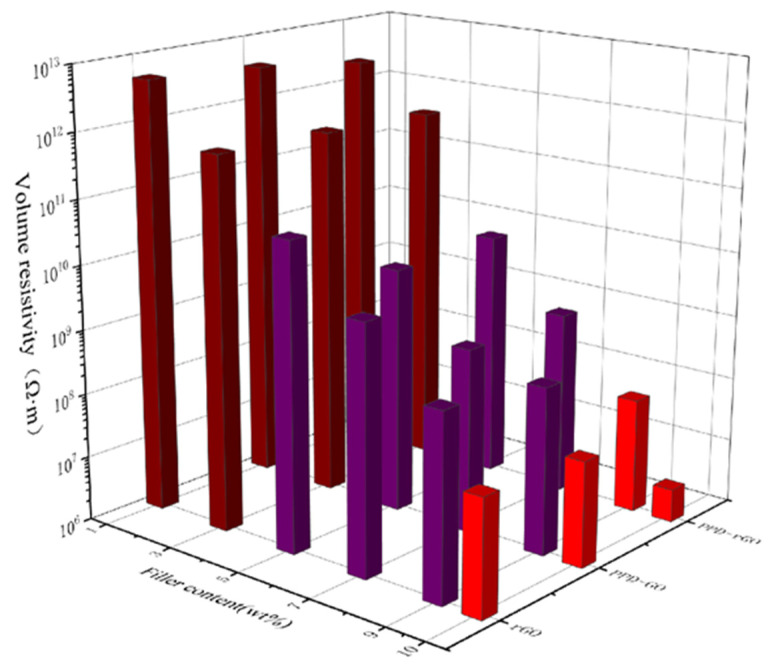
Volume resistivity diagram of composite material.

Figure 8 shows the volume resistivity of the composite material. The volume resistivity of the composite remains in the high insulation resistance range at a lower amount of filler. When the amount of filler reaches 5%, the volume resistivity decreases, reaching 10^9^ orders of magnitude, but it is still in the insulation range. When the filling amount reaches 9 wt%, the volume resistivity of PPD-rGO/EP reaches 6.05 × 10^7^ Ω·m, still in the insulation range, but near the permeability threshold. When the amount of filler reaches 10 wt%, the volume resistivity of PPD-rGO/EP reaches 3.26 × 10^6^ Ω·m, which is theoretically close to the conductor range.

## 4. Conclusions

In summary, GO was modified by p-phenylenediamine. The carboxyl group on GO was covalently amidated with the amino group on p-phenylenediamine to introduce the amino group, improving the compatibility of filler with epoxy resin. After that, the green mild reducing agent VC was used to reduce and eliminate the excess oxygen-containing groups on GO so as to repair its defects. Finally, a thermal conductivity of 1.7 W/mK and a volume resistivity of 6.05 × 10^7^ Ω·m were obtained by filling the epoxy resin with 9 wt% PPD-rGO. These met the requirements of for the heat conduction and insulation of electronic packaging materials and provided a new material for the heat conduction of electronic equipment. However, when the epoxy resin was filled with 10 wt% PPD-rGO, the thermal conductivity increased, but the volume resistivity still decreased. Thus, the further addition of PPD-rGO did not meet the insulation requirements of electronic packaging materials, and the filling amount of PPD-rGO of about 9 wt% was more appropriate. These results can be compared with recent achievements in related studies: for example, Han et al. [27] prepared composite materials with a highest thermal conductivity of 1.96 W/mK and high electromagnetic shielding performance by filling epoxy resin with 3D hybrid carbon nanostructures with covalent bonds. Fabiola et al. [28] achieved a thermal conductivity of 1.95 W/mK for the epoxy resin composite by filling 15 wt% graphenes and copper nanoparticle mixed filler. Sun et al. [29] modified graphenes through a polydopamine and silane coupling agent and the composite material prepared had a thermal conductivity of 0.73 W/mK, a low coefficient of thermal expansion (CTE), high thermal stability, low moisture absorption, and effective electrical insulation under a load of 5 wt%. The experimental results make a clear judgment on the influence of modified filler load on the thermal conductivity and insulation properties of the composite, making the PPD-rGO-filled epoxy resin composite a new type of thermal conductivity and electrical insulation material that potentially has application value in the electronic packaging industry.

## Figures and Tables

**Table 1:** Physicochemical characteristics of the matrix material at room temperature.

The Name of the Material	The Epoxy Value	Epoxy Equivalent	Viscosity	The Amine Value
Epoxy resin (E6002)	0.46–0.51 (eq/100 g)	185–210 (g/eq)	1100–1600 (mPas)	/
DETDA	/	/	280–300 (mPas)	620–640 (mg KOH/g)

**Table 2:** Characteristic thermal parameters of composite materials.

Samples	T_10%_ (°C)	Residue (%)
EP	358	4.65
PPD-rGO@EP-1%	365	7.03
PPD-rGO@EP-3%	360	7.93
PPD-rGO@EP-5%	366	8.70
PPD-rGO@EP-7%	368	9.05
PPD-rGO@EP-9%	370	10.22
PPD-rGO@EP-10%	364	10.43

## Data Availability

The data presented in this study are available on request from the corresponding author.

## References

[1] Pan C., Kou K.C., Jia Q., Zhang Y., Wu G.L., Ji T.Z. (2017). Improved thermal conductivity and dielectric properties of hBN/PTFE composites via surface treatment by silane coupling agent. Compos. Part B Eng..

[2] Huang X.Y., Jiang P.K., Tanaka T. (2011). A review of dielectric polymer composites with high thermal conductivity. IEEE Electr. Insul. Mag..

[3] Jiang J., Liu F.X., Zhuang K.Y., Chen D.Q., Chen G.H. (2017). Composites of epoxy/graphene-modified-diamond filler show enhanced thermal conductivity and high electrical insulation. RSC Adv..

[4] Chen L.F., Yu W., Xie H.Q. (2012). Enhanced thermal conductivity of nanofluids containing Ag/MWNT. composites Powder Technol..

[5] Chen W., Wang Z.F., Zhi C.Y., Zhang W.J. (2016). High thermal conductivity and temperature probing of copper nanowire/upconversion nanoparticles/epoxy composite. Compos. Sci. Technol..

[6] Mu Q.W., Li Q.M., Liu H. (2016). Enhancing the thermal conductivities of SiO_2_/Epoxy composites by orientation. Polym. Compos..

[7] Long J.J., Li C.X., Li Y. (2022). Enhancement of mechanical and bond properties of epoxy adhesives modified by SiO_2_ nanoparticles with active groups. Polymers.

[8] Zhang D.W., Liu F.S., Wang S., Yan M.X., Hu X., Xu M.Y. (2021). D-GQDs modified epoxy resin enhances the thermal conductivity of AlN/Epoxy resin thermally conductive composites. Polymers.

[9] Hu M.C., Feng J.Y., Ng K.M. (2015). Thermally conductive PP/AlN composites with a 3-D segregated structure. Compos. Sci. Technol..

[10] Shen H., Guo J., Wang H., Zhao N., Xu J. (2015). Bioinspired modification of h-BN for high thermal conductive composite films with aligned structure. ACS Appl. Mater. Interfaces.

[11] Yang D., Ni Y.F., Kong X.X., Gao D.H., Wang Y., Hu T.T., Zhang L.Q. (2019). Mussel-inspired modification of boron nitride for natural rubber composites with high thermal conductivity and low dielectric constant. Compos. Sci. Technol..

[12] Yu J., Choi H.K., Kim H.S., Kim S.Y. (2016). Synergistic effect of hybrid graphene nanoplatelet and multi-walled carbon nanotube fillers on the thermal conductivity of polymer composites and theoretical modeling of the synergistic effect. Compos. Part A Appl. Sci. Manuf..

[13] Du X.S., Liu H.Y., Mai Y.W. (2016). Ultrafast synthesis of multifunctional N-doped graphene foam in an ethanol flame. ACS Nano.

[14] Schedin F., Geim A.K., Morozov S.V., Hill E.W., Blake P., Katsnelson M.I., Novoselov K.S. (2007). Detection of individual gas molecules adsorbed on graphene. Nat. Mater..

[15] Gong J.R., Liu Z.D., Yu J.H., Dai D., Dai W., Du S.Y., Li C.Y., Jiang N., Zhan Z.L., Lin C.T. (2016). Graphene woven fabric-reinforced polyimide films with enhanced and anisotropic thermal conductivity. Compos. Part A Appl. Sci. Manuf..

[16] Lian G., Tuan C.C., Li L.Y., Jiao S.L., Wang Q.L., Moon K.S., Cui D.L., Wong C.P. (2016). Vertically aligned and interconnected graphene networks for high thermal conductivity of epoxy composites with ultralow loading. Chem. Mater..

[17] Alam F.E., Dai W., Yang M.H., Du S.Y., Li X.M., Yu J.H., Jiang N., Lin C.T. (2017). In situ formation of a cellular graphene framework in thermoplastic composites leading to superior thermal conductivity. J. Mater. Chem. A.

[18] Li R.J., Yang Y., Wu D.T., Li K.L., Qin Y., Tao Y.X., Kong Y. (2019). Covalent functionalization of reduced graphene oxide aerogels with polyaniline for high performance supercapacitors. Chem. Commun..

[19] Liu C., Dong Y.F., Lin Y., Yan H.X., Zhang W.B., Bao Y., Ma J.Z. (2019). Enhanced mechanical and tribological properties of graphene/bismaleimide composites by using reduced graphene oxide with non-covalent functionalization. Compos. Part B Eng..

[20] Wang X.D., Liu X.H., Yuan H.Y., Liu H., Liu C.T., Li T.X., Yan C., Yan X.R., Shen C.Y., Guo Z.H. (2018). Non-covalently functionalized graphene strengthened poly (vinyl alcohol). Mater. Des..

[21] Liu Y.C., Lu M.P., Wu K., Jiao E.X., Liang L.Y., Shi J., Lu M.E. (2021). Enhanced thermal conduction of functionalized graphene nanoflake/polydimethylsiloxane composites via thermoluminescence strategy. Compos. Sci. Technol..

[22] Jr W.S.H., Offeman R.E. (1958). Preparation of graphitic oxide. J. Am. Chem. Soc..

[23] Liu S., Yu B., Zhang T. (2013). Preparation of crumpled reduced graphene oxide–poly(p-phenylenediamine) hybrids for the detection of dopamine. J. Mater. Chem. A.

[24] Ding P., Zhang J., Song N., Tang S.F., Liu Y.M., Shi L.Y. (2015). Anisotropic thermal conductive properties of hot-pressed polystyrene/graphene composites in the through-plane and in-plane directions. Compos. Sci. Technol..

[25] Jing L.C., Wang T., Rhen D.S., Yuan X.T., Tian Y., Xie Q.X., Geng H.Z. (2021). Wrinkled p-phenylenediamine grafted graphene oxide as reinforcement for polyvinyl butyral anti-corrosive coating. J. Mater. Sci..

[26] Wazalwar R., Raichur A.M. (2021). Model-free cure kinetics of tetra-functional epoxy reinforced with GO and p-Phenylenediamine modified GO. Thermochim. Acta.

[27] Han L., Li K., Fu Y., Yin X., Jiao Y., Song Q. (2022). Multifunctional electromagnetic interference shielding 3D reduced graphene oxide/vertical edge-rich graphene/epoxy nanocomposites with remarkable thermal management performance. Compos. Sci. Technol..

[28] Lopez-Barajas F., Ramos-deValle L.F., Sanchez-Valdes S., Ramirez-Vargas E., Martinez-Colunga J.G., Espinoza-Martinez A.B., da Silva L., Hernandez-Gamez J.F., Rodriguez-Fernandez O.S., Beltran-Ramirez F.I. (2022). Epoxy/hybrid graphene-copper nanocomposite materials with enhanced thermal conductivity. J. Appl. Polym. Sci..

[29] Sun Z., Wong R., Yu M., Li J., Zhang M., Mele L., Hah J., Kathaperumal M., Wong C.P. (2022). Nanocomposites for future electronics device Packaging: A fundamental study of interfacial connecting mechanisms and optimal conditions of silane coupling agents for Polydopamine-Graphene fillers in epoxy polymers. Chem. Eng. J..

